# Antidiabetic and renoprotective effects of the chloroform extract of *Terminalia *chebula Retz. seeds in streptozotocin-induced diabetic rats

**DOI:** 10.1186/1472-6882-6-17

**Published:** 2006-05-07

**Authors:** Nalamolu Koteswara Rao, Srinivas Nammi

**Affiliations:** 1 Pharmacology Division, GITAM Dental College, Visakhapatnam 530045, Andhra Pradesh, India; 2 Pharmacology Division, Department of Pharmaceutical Sciences, Andhra University, Visakhapatnam 530003, Andhra Pradesh, India; 3 Section of Endocrinology and Metabolism, Department of Internal Medicine, University of Manitoba, Winnipeg R3E3P4, Canada

## Abstract

**Background:**

*Terminalia chebula *(Combretaceae) has been widely used in Ayurveda for the treatment of diabetes. In the present investigation, the chloroform extract of *T. chebula *seed powder was investigated for its antidiabetic activity in streptozotocin-induced diabetic rats using short term and long term study protocols. The efficacy of the extract was also evaluated for protection of renal functions in diabetic rats.

**Methods:**

The blood glucose lowering activity of the chloroform extract was determined in streptozotocin-induced (75 mg/kg, i.p.; dissolved in 0.1 M acetate buffer; pH 4.5) diabetic rats, after oral administration at the doses of 100, 200 and 300 mg/kg in short term study. Blood samples were collected from the eye retro-orbital plexus of rats before and also at 0.5, 1, 2, 4, 6, 8 and 12 h after drug administration and the samples were analyzed for blood glucose by using glucose-oxidase/peroxidase method using a visible spectrophotometer. In long term study, the extract (300 mg/kg) was administered to streptozotocin-induced diabetic rats, daily for 8 weeks. Blood glucose was measured at weekly intervals for 4 weeks. Urine samples were collected before the induction of diabetes and at the end of 8 weeks of treatments and analyzed for urinary protein, albumin and creatinine levels. The data was compared statistically using one-way ANOVA with post-hoc Dunnet's *t*-test.

**Results:**

The chloroform extract of *T. chebula *seeds produced dose-dependent reduction in blood glucose of diabetic rats and comparable with that of standard drug, glibenclamide in short term study. It also produced significant reduction in blood glucose in long term study. Significant renoprotective activity is observed in *T. chebula *treated rats. The results indicate a prolonged action in reduction of blood glucose by *T. chebula *and is probably mediated through enhanced secretion of insulin from the β-cells of Langerhans or through extra pancreatic mechanism. The probable mechanism of potent renoprotective actions of *T. chebula *has to be evaluated.

**Conclusion:**

The present studies clearly indicated a significant antidiabetic and renoprotective effects with the chloroform extract of *T. chebula *and lend support for its traditional usage. Further investigations on identification of the active principles and their mode of action are needed to unravel the molecular mechanisms involved in the observed effects.

## Background

Diabetes mellitus is a debilitating and often life-threatening disorder with increasing incidence throughout the world [1]. Diabetic complications arise partly from glycosylation damage to structural and functional proteins and reflect chronic failure to maintain blood glucose homeostasis. Other complications such as diabetic nephropathy, diabetic retinopathy, diabetic neuropathy and diabetic cardiomyopathy prevail as a result of hyperglycemia. A scientific investigation of traditional herbal remedies for diabetes may provide valuable leads for the development of alternative drugs and strategies. Alternatives are clearly needed for better management of diabetes because of high cost and poor availability of current therapies for many rural populations, particularly in developing countries. Diabetic nephropathy is one of the microvascular complications of diabetes. The pathophysiology involves an interaction between metabolic and hemodynamic factors. Metabolic factors include advanced glycation, increased formation of polyols and activation of protein kinase-C. Hemodynamic factors include systemic hypertension, intraglomerular hypertension and the role of vasoactive hormones, such as anglotensin II. Clinical course progresses from microalbuminuria to overt proteinuria and then to renal failure [2].

The field of herbal medicines research has been gaining significant importance in the last few decades and the demand to use natural products in the treatment of diabetes is increasing worldwide. The available literature shows that there are more than 400 plant species showing antidiabetic activity [3,4]. Although some of these plants have great reputation in Ayurveda, the indigenous Indian system of medicine, many remain to be scientifically established [5].

The dried ripe fruit of *Terminalia chebula *Retz. (Combretaceae), is used extensively in Ayurveda and is widely distributed throughout India, Burma and Sri Lanka. It is commonly known as black myroblans in English and has traditionally been used in the treatment of asthma, sore throat, vomiting, hiccough, diarrhoea, bleeding piles, gout and heart and bladder diseases [6]. A herbal formulation containing *T. chebula *under the name 'TRIPHALA' is a very popular traditional medicine for the treatment of chronic disorders including diabetes [7,8]. It is reported to have antioxidant and free radical scavenging activities [9]. It has shown effectiveness against cancer cells [10] and helicobacter pylori [11]. It is also useful as anticaries agent [12] in dermal wound healing [13], improving gastrointestinal motility [14] and anaphylactic shock [15]. The methanolic extract of *T. chebula *has been shown to exhibit antidiabetic activity in rats [16]. Although the fruits are known for their antidiabetic properties, the whole powder of dried ripe fruits is also being widely used for the control of diabetes. So far, little is known on the medicinal values of *T. chebula *seeds. In the present study, the chloroform extract of the seeds of *T. chebula *was tested for its antidiabetic activity using short term and long term study protocols after oral administration in streptozotocin-induced diabetic rats. Moreover, the extract was also tested for its renoprotective effects upon long term study in diabetic rats.

## Methods

### Plant material and extraction

Fresh seeds of *T. chebula *were purchased from the local traders and shade dried to obtain a completely dried product. An authenticated voucher specimen (No. SP-26) of the plant has been preserved in our Department for future reference. The dried seeds were then milled to fine powder (3 kg) and extracted with chloroform in Soxhlet's apparatus for 24 h and the extract was evaporated to dryness under vacuum and dried in vacuum desiccator (270.8 g).

### Chemicals used

Glibenclamide was a generous gift sample from Sun Pharmaceuticals Limited, Baroda, India and streptozotocin was purchased from Sigma-Aldrich, St. Louis, USA. Glucose, albumin, creatinine and protein assay kits were obtained from the Diagnostic Division of Dr. Reddy's Laboratories, Hyderabad, India. All other chemicals used were of analytical grade.

### Animal experiments

Male Sprague Dawley rats procured from Mahaveer Enterprises, Hyderabad, India were used in the studies. All the animal experiments were conducted according to the protocols approved by the Institutional Animal Ethics Committee (Reg. No. 516/01/A/CPCSEA). Animals were divided into 8 groups of five each and were fed with standard diet (Ratan Brothers, Hyderabad) and water *ad libitum*. They were kept in clean and dry cages and maintained in well-ventilated animal house with 12 h light-12 h dark cycle. Rats were rendered diabetic by injecting a freshly prepared streptozotocin (75 mg/kg, i.p.; dissolved in 0.1 M acetate buffer; pH 4.5) [17] after a base-line blood glucose estimation was done. After two weeks, animals with blood glucose levels above 450 mg/dl were selected for the study.

### Short term studies

Groups I, II and III were given orally, the *T. chebula *extract in the form of suspension in 1% sodium CMC at doses of 100, 200 and 300 mg/kg respectively. Group IV was served as control and received equivalent volumes of vehicle. Group V received glibenclamide at a dose of 0.04 mg/kg and served as standard. Animals were fasted for 16 h prior to drug administration allowing access only to water. Blood samples were collected from retro-orbital plexus of each rat before and also at 0.5, 1, 2, 4, 6, 8 and 12 h after treatments. The samples were analyzed for blood glucose content by using glucose-oxidase/peroxidase method [18,19] with optical density measured at 505 nm using a visible spectrophotometer.

### Long term studies

For long term evaluation, groups of rats were given daily treatments for 8 weeks. Animals in group VI were given orally, the *T. chebula *extract (suspended in 1% sodium CMC) at a dose of 300 mg/kg. Group VII served as control while group VIII received glibenclamide at a dose of 0.04 mg/kg. Blood samples were collected from the overnight fasted animals and fasting blood glucose levels were measured before and also at weekly intervals for 4 weeks. For urinary collection, rats were housed in metabolic cages at the start (pre-diabetic condition) and at the end (8^th ^week) of the experiment. The 24 h urinary samples were collected from all the animals after have been acclimatized in metabolic cages for 3 days. The measurements of urinary protein [20], albumin [21] and creatinine [22] were done using commercial diagnostic kits following manufacturer's instructions.

### Statistical analysis

Data are expressed as mean ± standard error of mean. Statistical analysis was done using one-way analysis of variance (ANOVA) and post-hoc comparisons were carried out using Dunnet's *t*-test. P values <0.05 were considered as significant.

## Results

### Short term studies

The chloroform extract of *T. chebula *seeds produced significant antidiabetic effect with various doses in streptozotocin-induced diabetic rats in acute study. It produced a dose-dependent reduction in blood glucose with doses of 100, 200 and 300 mg/kg compared to control group (Table 1). Glibenclamide (0.04 mg/kg) also produced a significant reduction in blood glucose compared to control group. *T. chebula *produced a maximum reduction of blood glucose of 20.85% (p < 0.01), 28.45% (p < 0.001) and 42.20% (p < 0.001) at 4 h with doses of 100, 200 and 300 mg/kg respectively, where as glibenclamide (0.04 mg/kg) produced a maximum reduction of 50.44% (4 h, p < 0.001) compared to control group.

**Table 1:** Percent blood glucose reduction produced by *T.chebula *after oral administration in streptozotocin-induced diabetic rats.

**Group (n = 5)**	**Dose (mg/kg)**	**Percent blood glucose reduction**						
		
		**0.5**	**1**	**2**	**4**	**6**	**8**	**12 (hrs)**
Control	---	-5.65 ± 1.80	-8.10 ± 3.29	-1.35 ± 6.62	-12.09 ± 4.48	-7.25 ± 4.90	-4.19 ± 2.08	-7.03 ± 8.15
*T.chebula*	100	-2.18 ± 3.95	8.45 ± 9.27	17.82 ± 5.38*	20.85 ± 5.29*	19.94 ± 6.18*	10.98 ± 8.54	8.36 ± 3.18
*T.chebula*	200	1.28 ± 4.72	20.28 ± 6.29*	31.98 ± 8.24**	28.45 ± 6.20**	26.55 ± 2.70**	18.25 ± 5.21*	12.86 ± 8.20*
*T.chebula*	300	-1.18 ± 4.25	25.88 ± 10.05**	38.92 ± 7.18**	42.20 ± 4.65**	33.09 ± 8.05**	27.85 ± 4.78**	22.90 ± 7.55**
Glibenclamide	0.04	24.92 ± 8.20*	32.37 ± 4.60**	38.72 ± 10.22**	50.44 ± 8.40**	46.56 ± 4.60**	23.11 ± 6.75*	10.26 ± 11.03

Values are represented as mean ± SEM.; n = number of animals per group

Significant difference from control group at identical times, *P < 0.01; **P < 0.001

### Long term studies

Long term administration of *T. chebula *(300 mg/kg) to streptozotocin-induced diabetic rats for four weeks produced significant reduction in blood glucose. The reduction was significant after treatment for one week in both the extract and glibenclamide treated groups and continued to increase up to four weeks (Table 2). At the end of 4^th ^week, *T. chebula *extract produced significant blood glucose reduction of 53.09% (p < 0.01). On the other hand, glibenclamide produced significant blood glucose reduction of 60.10% (p < 0.01).

**Table 2:** Percent blood glucose reduction produced by *T. chebula *after chronic administration in streptozotocin-induced diabetic rats

**Group (n = 5)**	**Dose (mg/kg)**	**Percent blood glucose reduction**			
		
		**1**	**2**	**3**	**4 (Weeks)**
Control	----	-19.72 ± 8.05	-14.35 ± 4.92	-7.06 ± 4.28	-12.48 ± 7.55
*T. chebula*	300	38.75 ± 8.09*	45.20 ± 11.64*	48.10 ± 5.25*	53.09 ± 3.14*
Glibenclamide	0.04	48.09 ± 10.90*	55.75 ± 8.03*	58.88 ± 5.08*	60.10 ± 2.70*

Values are represented as mean ± S.E.M.; n = number of animals per group

Significant difference from control group at identical times, *P < 0.01

**Figure 1:**
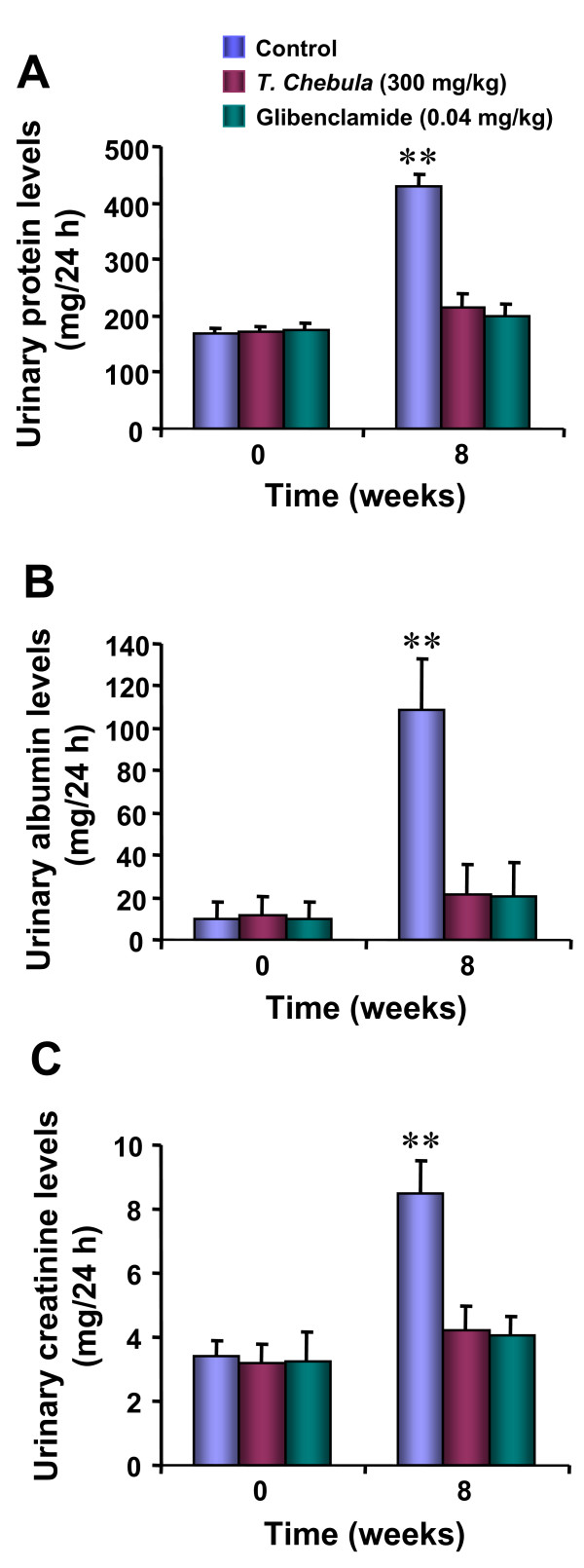
**Effect of T.*chebula *on urinary excretion of protein (A), albumin (B) and creatinine (C) in streptozotocin-induced diabetic rats**. Each bar indicates the mean ± SEM of 5 animals in each group. Urine samples were collected before the induction of diabetes (0 week) and after 8 weeks of daily treatments either with vehicle (control), *T. chebula *(300 mg/kg) or glibenclamide (0.04 mg/kg) in streptozotocin-induced diabetic rats. Significant difference from the corresponding pre-diabetic value: **p < 0.001.

The urinary excretion profile of protein, albumin and creatinine is shown in Figure 1. Streptozotocin-induced diabetic rats displayed a significant (p < 0.001) increase in urinary protein, albumin and creatinine after 8 weeks as compared to their pre-diabetic state levels. However, rats treated with *T. chebula *(300 mg/kg) or glibenclamide (0.04 mg/kg) for 8 weeks did not produce any change in urinary protein, albumin or creatinine compared to their corresponding pre-diabetic values.

## Discussion

Diabetes mellitus is possibly the World's largest growing metabolic disorder, and as the knowledge on the heterogeneity of this disorder is advanced, the need for more appropriate therapy increases [23]. The enormous costs of modern medicines indicate that alternative strategies are required for better management of diabetes. Traditional plant medicines are used throughout the world for a range of diabetic complications. The study of such medicines might offer a natural key to unlock a diabetologist's pharmacy for the future.

For the study of antidiabetic agents, streptozotocin-induced hyperglycemia in rodents is considered to be a good preliminary screening model and is widely used [24]. Streptozotocin, N- [methylnitrocarbamoyl]-D-glucosamine is a potent methylating agent for DNA and acts as nitric oxide donor in pancreatic β-cells and thus β-cells are more sensitive to damage by nitric oxide and free radical scavenging enzymes [25]. *T. chebula *is widely used as a traditional medicine by diabetic patients in India. Although the fruits are known for their antidiabetic properties, the whole powder of dried ripe fruits is also being widely used for the control of diabetes. So far, little is known on the medicinal values of *T. chebula *seeds. In the present study the chloroform extract of the seeds of *T. chebula *was evaluated and the data also confirmed the traditional indications. Our data on the seed extract of *T. chebula *indicated a potent action in short term study and a prolonged duration of antidiabetic action in long term study and this could be due to multiple sites of action possessed by the active principles of *T. chebula*.

The study also revealed that *T. chebula *is more effectively inhibited the incidence of diabetic nephropathy. Diabetic nephropathy is mainly associated with excess urinary albumin excretion, abnormal renal function as represented by an abnormality in serum creatinine. The common progression from microalbuminuria to overt nephropathy has led many to consider microalbuminuria to define early or incipient nephropathy. Renal disease is suspected to be secondary to diabetes in the clinical setting of long-standing diabetes. Clinically, diabetic nephropathy is characterized by a progressive increase in proteinuria and decline in GFR, hypertension, and a high risk of cardiovascular morbidity and mortality. The pathophysiology involves glucose that binds irreversibly to proteins in the kidney and circulation to form advanced glycosylation end products (AGEs). AGEs can form complex crosslinks over years of hyperglycemia and can contribute to renal damage by stimulation of growth and fibrotic factors via receptors for AGEs. Increased glomerular capillary pressure occurs early in diabetes and is associated with hyperfiltration at the glomerulus. The glomerular mesangium expands, initially by cell proliferation and then by cell hypertrophy. Increased mesangial stretch and pressure can stimulate this expansion, as can high glucose levels. Mediators of proliferation and expansion include platelet-derived growth factor and transforming growth factor-β (TGF-β). TGF-β are particularly important in the mediation of expansion and later fibrosis *via *the stimulation of collagen and fibronectin. Angiotensin-II (AT-II) also contributes to the progression of diabetic nephropathy. AT-II preferentially constricts the efferent arteriole in the glomerulus, leading to higher glomerular capillary pressure. In addition to its hemodynamic effects, AT-II also stimulates renal growth and fibrosis through AT-II type 1 receptors, which secondarily upregulate TGF-β and other growth factors. The extract due to its significant hypoglycemic activity may have inhibited the formation of advanced glycosylation end products. However the extract may also have effect on the above stated other mechanisms.

Further work on fractionation, purification, identification of active principle(s) and detailed mechanistic evaluation responsible for these activities are obviously required on the seeds of *T. chebula*. The possible mechanisms behind the hypoglycemic activity and the inhibition of incidence of diabetic nephropathy are yet to be studied.

## Conclusion

In conclusion, the present studies indicated a significant antidiabetic and renoprotective effects with the chloroform extract of *Terminalia chebula *and support its traditional usage in the control of diabetes and its complications. Further investigations to identify the active principle(s) are obviously needed together with a detailed evaluation on the mechanisms involved in the observed activities.

## Competing interests

The author(s) declare that they have no competing interests.

## Authors' contributions

NKR conceived the study, made significant contributions in data analysis, data interpretation, writing of the manuscript and in coordination of the experiments. SN made substantial contributions in conceptualization of statistical analyses, drafting the final manuscript and designing the illustrations. All authors read and approved the final manuscript.

## Pre-publication history

The pre-publication history for this paper can be accessed here:


